# Beyond Current Borders: Modeling the Climate‐Mediated Invasion of *Fusarium incarnatum* Into New Agricultural Frontiers

**DOI:** 10.1002/ece3.74057

**Published:** 2026-07-26

**Authors:** Jawaher Ibrahim Alahadeb

**Affiliations:** ^1^ Department of Biology, College of Science (Majmaah) Majmaah University ALmajmaah Saudi Arabia

**Keywords:** bioclimatic variables, climate change, *Fusarium incarnatum*, MaxEnt modeling, plant pathogen, species distribution modeling

## Abstract

Climate change is altering the global distribution of plant diseases, significantly impacting agricultural sustainability and food security. *Fusarium incarnatum*, an economically significant fungal pathogen responsible for substantial crop losses, poses a serious threat, yet its distribution dynamics under shifting climatic conditions are inadequately understood. This research utilized Maximum Entropy (MaxEnt) modeling integrated with Geographic Information Systems (GIS) to evaluate the present and future global distribution of *F. incarnatum* under climate change scenarios. Species occurrence data (*n* = 112) were acquired from the Global Biodiversity Information Facility (GBIF) using stringent spatial filtering. Five bioclimatic variables were identified via correlation analysis and significance evaluation: Annual Mean Temperature (bio1), Mean Diurnal Range (bio2), Isothermality (bio3), Mean Temperature of the Warmest Quarter (bio10), and Precipitation of the Driest Month (bio14). Future projections for 2050 and 2070 were generated using Representative Concentration Pathways (RCP) 2.6 and 8.5, based on ensemble means from three Global Circulation Models. The MaxEnt model demonstrated outstanding predictive performance (AUC = 0.88, TSS = 0.80), with Annual Mean Temperature identified as the principal limiting factor. Current suitable habitats are located in temperate and subtropical regions across all continents, with consensus zones between MaxEnt and Bioclim models indicating the most reliable forecasts. Future projections reveal a concerning northward range expansion, with extensive areas of Canada, northern Europe, and Siberia transitioning from unsuitable to medium‐high suitability by 2050–2070. The RCP 8.5 scenario projected a more extensive expansion than RCP 2.6, underscoring the critical importance of climate mitigation. Factor analysis indicated temperature‐related constraints in northern latitudes and moisture restrictions in semi‐arid zones. These findings provide critical insights for developing early warning systems, targeted surveillance, and adaptive management strategies to mitigate the agricultural risks associated with the climate‐driven expansion of *F. incarnatum*, thereby supporting global food security planning.

## Graphical abstract

This study models the global distribution of *Fusarium incarnatum* under current and future climate scenarios using MaxEnt and Bioclim approaches. Model projections identify regions of high suitability across temperate and subtropical zones, with consensus between modeling methods indicating the most reliable predictions. The findings reveal significant northward range expansion under climate change, highlighting emerging agricultural risks in previously unsuitable regions.

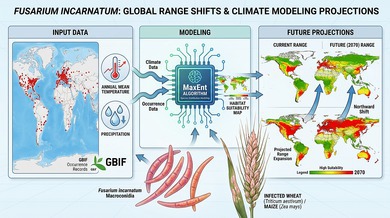

## 1 Introduction

Anthropogenic climate change represents one of the most pressing challenges of the 21st century, with cascading effects on biodiversity conservation, ecosystem functioning, and agricultural productivity (Anderegg et al. 2015). Alterations in global temperature and precipitation regimes are fundamentally reshaping the geographic distributions and population dynamics of organisms across all taxonomic groups, including fungi that pose significant threats to food security and human welfare (Bebber et al. 2013; Chaloner et al. 2021). Range shifts in response to changing environmental conditions have emerged as a critical research frontier, particularly for understanding future distributions of economically important pathogens.

Fungal pathogens occupy pivotal roles in terrestrial ecosystems and display marked sensitivity to climatic variation due to their dependence on specific temperature and moisture regimes for growth, sporulation, and dispersal (Garrett et al. 2006; Chakraborty et al. 2000). The genus *Fusarium* encompasses numerous species recognized as highly destructive plant pathogens worldwide, causing substantial economic losses in agriculture through diverse disease syndromes including wilts, blights, and root rots (Yadav et al. 2017). As plant pathogens, *Fusarium* species are notorious for inducing crop diseases such as blight, root and stem rots, and vascular wilts, establishing them as the most prominent mycotoxigenic genus affecting both weedy and cultivated plants across diverse climatic zones globally (Abdel‐Rhman et al. 2023). These diseases not only compromise crop yield and quality but also result in mycotoxin contamination that poses serious health risks to humans and livestock, making their investigation essential for both agricultural and public health considerations.

*Fusarium incarnatum* represents a particularly significant species within this genus, exhibiting worldwide distribution and causing substantial yield reductions in numerous agricultural systems (Lal et al. 2022). This species belongs to the *Fusarium incarnatum‐equiseti* species complex (FIESC), which is characterized by considerable pathogenic potential and prevalence across various cropping systems (Bergna et al. 2018). The pathogen displays remarkable adaptability to diverse climatic conditions and host plant species, rendering it a formidable threat to global food security. Recent reports have documented the emergence of this pathogen in novel geographical regions, suggesting that climate change may be facilitating its expansion into previously unsuitable areas.

The relationship between climate change and fungal pathogen range expansion has become increasingly apparent, with mounting evidence suggesting that altered climatic conditions are creating novel opportunities for disease establishment and spread (Valdes et al. 2024). Climate change is creating conditions conducive to the emergence of novel fungal diseases in new geographic regions while enabling fungi to adapt to previously inhospitable environments, including contaminated habitats and urban areas, resulting in the geographic spread of certain fungi to regions that were historically unsuitable (Bebber 2015; Bebber et al. 2013). Rising temperatures, altered precipitation patterns, and extreme weather events are fundamentally transforming the ecological niches available to fungal pathogens, potentially expanding their geographic ranges and intensifying their impacts on agricultural systems. The enhanced thermotolerance observed in many fungal species under changing climatic conditions may increase their virulence and fitness, potentially amplifying their pathogenic effects (McDonald and Stukenbrock 2016; Schoustra et al. 2007).

Species distribution modeling has emerged as a powerful tool for understanding and predicting potential impacts of climate change on organismal distributions (Elith et al. 2011). Among various modeling approaches, Maximum Entropy (MaxEnt) modeling has gained widespread adoption due to its robust performance and ability to utilize presence‐only data, often the only type of occurrence information available for many species (Phillips et al. 2006). Integration of MaxEnt modeling with Geographic Information Systems (GIS) provides a powerful framework for analyzing spatial patterns and projecting future distributions under alternative climate scenarios (Liang et al. 2023). This approach has proven particularly effective for fungal pathogens, where traditional survey methods may be limited by the cryptic nature of many species and the challenges associated with their detection and identification.

The application of bioclimatic variables in species distribution modeling has revolutionized our understanding of environmental factors that regulate species distributions (Yuan et al. 2019). These variables, derived from monthly temperature and precipitation values, represent biologically meaningful measures of annual trends, seasonality, and extreme or limiting environmental factors. MaxEnt is a machine learning‐based tool for species distribution modeling, widely used to estimate the probability of a species' presence. The algorithm's properties require appropriate selection of bioclimatic variables to ensure the model accurately predicts species occurrence. Bioclimatic variables can help explain the environmental constraints affecting distributions of fungal pathogens like *Fusarium incarnatum* and the potential for their range expansion under future climate scenarios. Identifying key bioclimatic factors associated with pathogen distribution is crucial for developing effective management strategies and early warning systems for disease outbreaks.

Understanding the global distribution patterns of *Fusarium incarnatum* and the bioclimatic factors influencing its occurrence is critical for developing proactive management strategies and assessing potential risks posed by this pathogen under changing climatic conditions. Previous studies on related *Fusarium* species have demonstrated the utility of species distribution modeling for understanding pathogen ecology and predicting future distributions (Abdel‐Rhman et al. 2023). *F. oxysporum*, a fascinating plant pathogen, has a cosmopolitan distribution, being found on all continents except Antarctica, making it a dynamic biological system for investigating the impacts of climate change. Nevertheless, comprehensive global analyses of *F. incarnatum* distribution and its relationship with climatic variables remain scarce, despite growing recognition of this pathogen's importance in agricultural systems worldwide.

This study aims to assess the impact of climate change on the global distribution of *Fusarium incarnatum* using MaxEnt modeling combined with GIS analysis and to identify critical bioclimatic variables governing current and future distribution patterns of this important plant pathogen. By developing robust predictive models using current species occurrence data and environmental variables, this research seeks to generate valuable insights into the potential geographic expansion of *F. incarnatum* under various climate change scenarios, thereby contributing to the development of more effective disease management strategies and food security planning in a changing world.

## 2 Materials and Methods

### 2.1 Occurrence Data

Species occurrence records for *Fusarium incarnatum* were obtained from the Global Biodiversity Information Facility (GBIF) database (www.gbif.org), which serves as the primary global repository for biodiversity information (Yesson et al. 2007). Initially, 2233 occurrence points were retrieved for modeling purposes. To ensure data quality and minimize spatial bias, the dataset was subjected to a rigorous three‐step filtering procedure following established best practices for species distribution modeling (Boria et al. 2014; Alqahtani, Shahin, et al. 2025).

The first step involved the removal of occurrence records lacking precise geographic coordinates (longitude and latitude information). Subsequently, duplicate records were identified and eliminated to prevent overrepresentation of specific localities. Spatial rarefaction was performed using a 10 km distance threshold in ArcGIS v. 10.3 (SDM toolbox: SDM tools; Universal tools—Spatially rarefy occurrence data) to reduce spatial autocorrelation and sampling bias (Brown et al. 2017). The rarefaction procedure is crucial for preventing model overfitting and ensuring that environmental space is adequately sampled without bias toward densely sampled areas. A 10 km threshold was selected as it exceeds the spatial grain of the bioclimatic layers (~5 km at 2.5 arc‐minutes), ensuring that retained records represent environmentally distinct localities and are consistent with thresholds applied in comparable global‐scale fungal SDM analyses (Alkhalifah et al. 2022; Alqahtani, Shahin, et al. 2025). After applying these filtering criteria, 112 unique occurrence points were retained and exported to CSV format for subsequent analysis in current and future potential distribution models of *F. incarnatum*.

### 2.2 Climate Data

Environmental data consisted of 19 bioclimatic variables representing temperature and precipitation patterns, obtained from the WorldClim database (www.worldclim.org) at a spatial resolution of 2.5 arc‐minutes (approximately 5 km^2^) (Fick and Hijmans 2017). These variables represent monthly climatic data averaged over the period 1950–2000 from weather station records, and provide biologically meaningful measures of annual trends, seasonality, and extreme environmental conditions that influence species distributions (Hijmans et al. 2005; Khalaf et al. 2024a).

Variable selection was performed through correlation analysis and assessment of variable importance to optimize model performance and reduce computational complexity. Bioclimatic variables 8–9 and 18–19 were initially excluded due to spatial anomalies that could potentially compromise model accuracy, following recommendations from previous studies (Escobar et al. 2014; Merow et al. 2013). The remaining 15 variables were converted to ASCII format using ArcGIS v. 10.3 to ensure compatibility with modeling software.

Multicollinearity among environmental variables was addressed by calculating Pearson's correlation coefficients, with subsequent removal of variables showing high correlation (*r* ^2^ > |0.8|) to avoid redundancy in the modeling process (Dormann et al. 2013). The SDM toolbox functionality in ArcGIS v. 10.3 (Universal tool; Explore climate data; Remove highly correlated variables) was employed to systematically address multicollinearity (Brown et al. 2017). Five key bioclimatic variables were selected for final analysis based on correlation analysis and variable importance scores from preliminary MaxEnt runs: Bio_1 (Annual Mean Temperature), Bio_2 (Mean Diurnal Range), Bio_3 (Isothermality), Bio_10 (Mean Temperature of the Warmest Quarter), and Bio_14 (Precipitation of the Driest Month).

Future climate projection datasets for bioclimatic variables were obtained from WorldClim for time periods 2041–2060 (2050s) and 2061–2080 (2070s) under Representative Concentration Pathways (RCPs) 2.6 and 8.5 (van Vuuren et al. 2011). RCP 2.6 represents a low emission scenario with strong mitigation policies, while RCP 8.5 represents a high emission scenario with minimal climate mitigation (Meinshausen et al. 2011). Three General Circulation Models (GCMs) were employed for both RCPs: BCC‐CSM1.1 (Beijing Climate Center), CCSM4 (National Center for Atmospheric Research), and MRI‐CGCM3 (Meteorological Research Institute). Ensemble means of the three GCMs were calculated for each RCP and time period to reduce uncertainty associated with individual model projections, following established climate projection protocols (Araújo and New 2007; Alenezi et al. 2026).

### 2.3 Modeling Approach

The Maximum Entropy (MaxEnt) software package version 3.4.1 was used to model current and future potential distributions of *F. incarnatum* (Phillips et al. 2006). MaxEnt is a machine‐learning algorithm that estimates species distributions by finding the maximum entropy probability distribution constrained by environmental data at occurrence localities (Elith et al. 2011). The method is particularly suitable for presence‐only data and has been extensively validated for predicting species distributions across diverse taxonomic groups (Merow et al. 2013; Alkhalifah et al. 2022).

The modeling procedure utilized 75% of occurrence records for model training, with the remaining 25% reserved for independent testing, following standard protocols for species distribution modeling (Fielding and Bell 1997). Background points were set at 10,000 to adequately represent environmental space, and maximum iterations were set to 500 to ensure model convergence. To enhance model robustness and quantify prediction uncertainty, 10‐fold cross‐validation was implemented, whereby the dataset was randomly divided into 10 subsets, with each subset used once for testing while the remaining nine were used for training (Pearson et al. 2007).

Resulting habitat suitability maps represent relative probability of occurrence (habitat suitability index, 0–1), which was classified into five interpretable categories based on probability thresholds following established protocols (Peterson et al. 2011): unsuitable (0–0.2), low suitability (0.2–0.4), medium suitability (0.4–0.6), high suitability (0.6–0.8), and very high suitability (0.8–1.0), using ArcGIS v. 10.3. Future distribution changes were analyzed by assessing habitat gain and loss using the map algebra function in ArcGIS v. 10.3 (Khalaf et al. 2024b).

### 2.4 Model Evaluation

Model performance was assessed using two complementary metrics: the Area Under the Curve (AUC) of the Receiver Operating Characteristic (ROC) and the True Skill Statistic (TSS) (Fielding and Bell 1997; Khalaf et al. 2025). AUC values range from 0 to 1, with values below 0.5 indicating performance no better than random, values between 0.5 and 0.7 indicating poor performance, values from 0.7 to 0.9 indicating good performance, and values exceeding 0.9 indicating excellent model performance (Swets 1988). TSS values range from −1 to +1, with values near 0 indicating poor model performance and values approaching 1 indicating excellent model performance with perfect discrimination between presence and absence (Allouche et al. 2006).

Statistical significance of model performance was evaluated using the binomial test of omission, which compares the proportion of test localities within predicted suitable areas against the null hypothesis of random distribution (Pearson et al. 2007). Additionally, jackknife tests were performed to assess the importance of each environmental variable in contributing to model performance.

### 2.5 Envelope Testing and Limiting Factor Analysis

Two‐dimensional niche analysis was conducted using DIVA‐GIS software to examine the relationship between *F. incarnatum* occurrence and key environmental variables (Hijmans et al. 2012). Envelope testing was applied to Annual Mean Temperature (Bio_1) and Annual Precipitation (Bio_12) to define the two‐dimensional ecological niche of the species and determine optimal climatic conditions for its occurrence (Hijmans et al. 2012; Alqahtani, Elshahawi, and Khalaf 2025).

Additionally, limiting factor maps were generated using the statistical modeling capabilities of DIVA‐GIS to identify the most constraining environmental variables across the study area (Hijmans et al. 2012). This analysis identifies which climatic factors most strongly limit species distribution in different geographic regions, providing insights into the mechanistic basis of species‐environment relationships and potential range shifts under climate change scenarios (Khalaf et al. 2026).

## 3 Results

### 3.1 Input Data Characteristics and Model Performance

#### 3.1.1 Input Data Characteristics and Taxonomic Considerations

**FIGURE 1:**
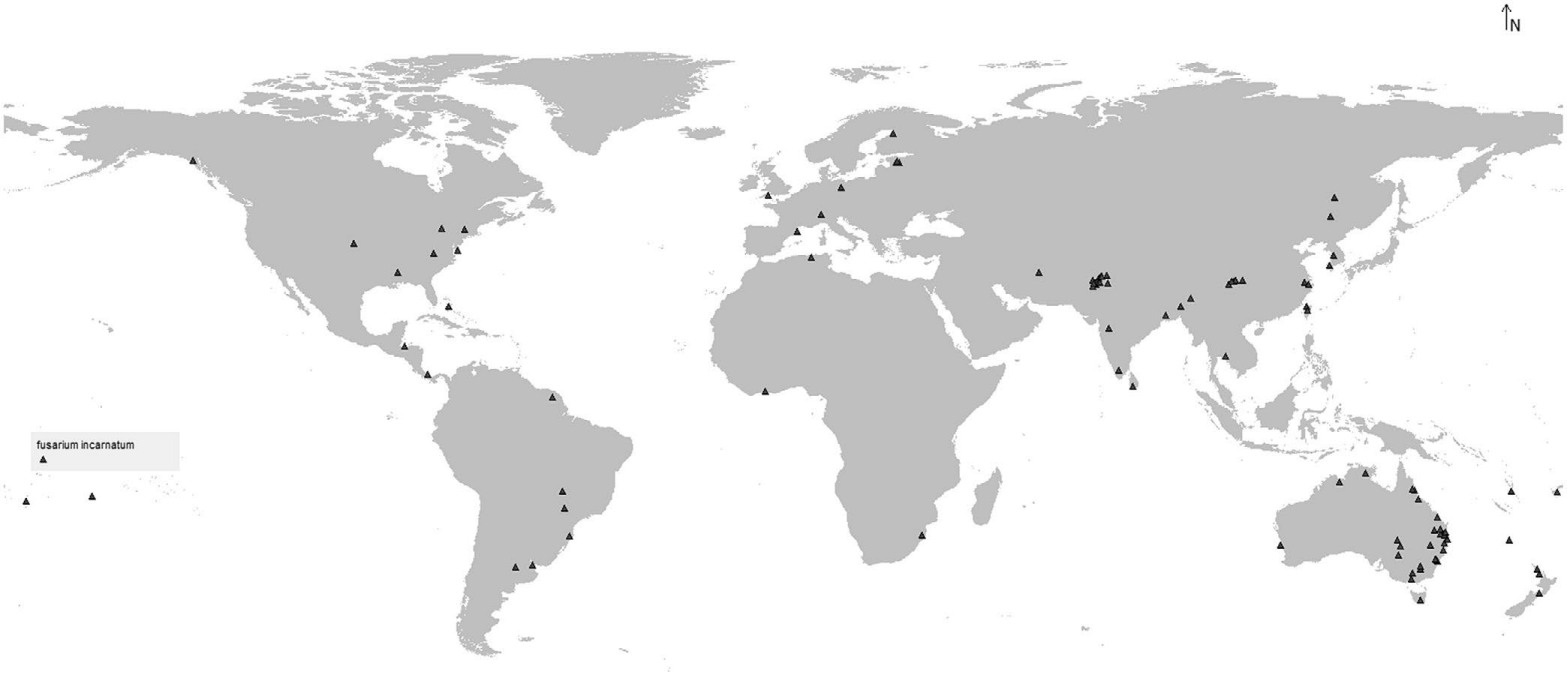
Geographic distribution of occurrence records for *Fusarium incarnatum* used in species distribution modeling. Points represent the 112 spatially rarefied occurrence locations obtained from the Global Biodiversity Information Facility (GBIF) after application of quality filtering and spatial autocorrelation reduction procedures. Some apparently isolated points in oceanic areas correspond to coastal or island localities (e.g., Caribbean islands, Pacific atolls) that appear separated from continental landmasses at the global map scale. Coordinate system: WGS 1984.

The final dataset comprised 112 spatially rarefied occurrence records for *Fusarium incarnatum* distributed across temperate and subtropical regions globally (Figure 1). These records span multiple continents including North America, Europe, Asia, South America, Africa, and Australia, with notable clustering in well‐surveyed regions of North America, Europe, and East Asia.

It is important to acknowledge that GBIF occurrence records for *F. incarnatum* are primarily based on ITS rDNA sequence data, which has well‐documented limitations in distinguishing closely related species within the *Fusarium incarnatum*‐*equiseti* species complex (FIESC). The FIESC encompasses over 30 cryptic phylogenetic species (phylo‐species), many of which remain unnamed and cannot be reliably differentiated using ITS sequences alone due to high sequence similarity (98%–100%) among members (O'Donnell et al. 2013; O'Donnell et al. 2020). Recent multi‐locus phylogenetic studies using calmodulin, RPB1, RPB2, and TEF‐1α genes have resolved these cryptic lineages, but GBIF data largely predates these advances (O'Donnell et al. 2013; Sarver et al. 2011). Consequently, the occurrence data used in this study likely represent an aggregate of multiple FIESC phylo‐species rather than a single taxonomic entity. This taxonomic uncertainty represents a significant caveat that is discussed further in the Discussion section.

#### 3.1.2 Model Performance and Bioclimatic Variables

The species distribution model for *Fusarium incarnatum* demonstrated excellent predictive performance, as evidenced by an Area Under the Curve (AUC) value of 0.88 and a True Skill Statistic (TSS) of 0.80, indicating substantial model accuracy and reliability in predicting the pathogen's potential distribution. These evaluation metrics demonstrate that the model effectively captures the environmental requirements of *F. incarnatum* and provides robust predictions suitable for ecological and agricultural applications. The high AUC value indicates strong discriminatory ability between suitable and unsuitable habitats, while the TSS value confirms that the model performs significantly better than random prediction, thus providing a solid foundation for understanding the pathogen's ecological niche and potential distribution under current and future climatic conditions.

**FIGURE 2:**
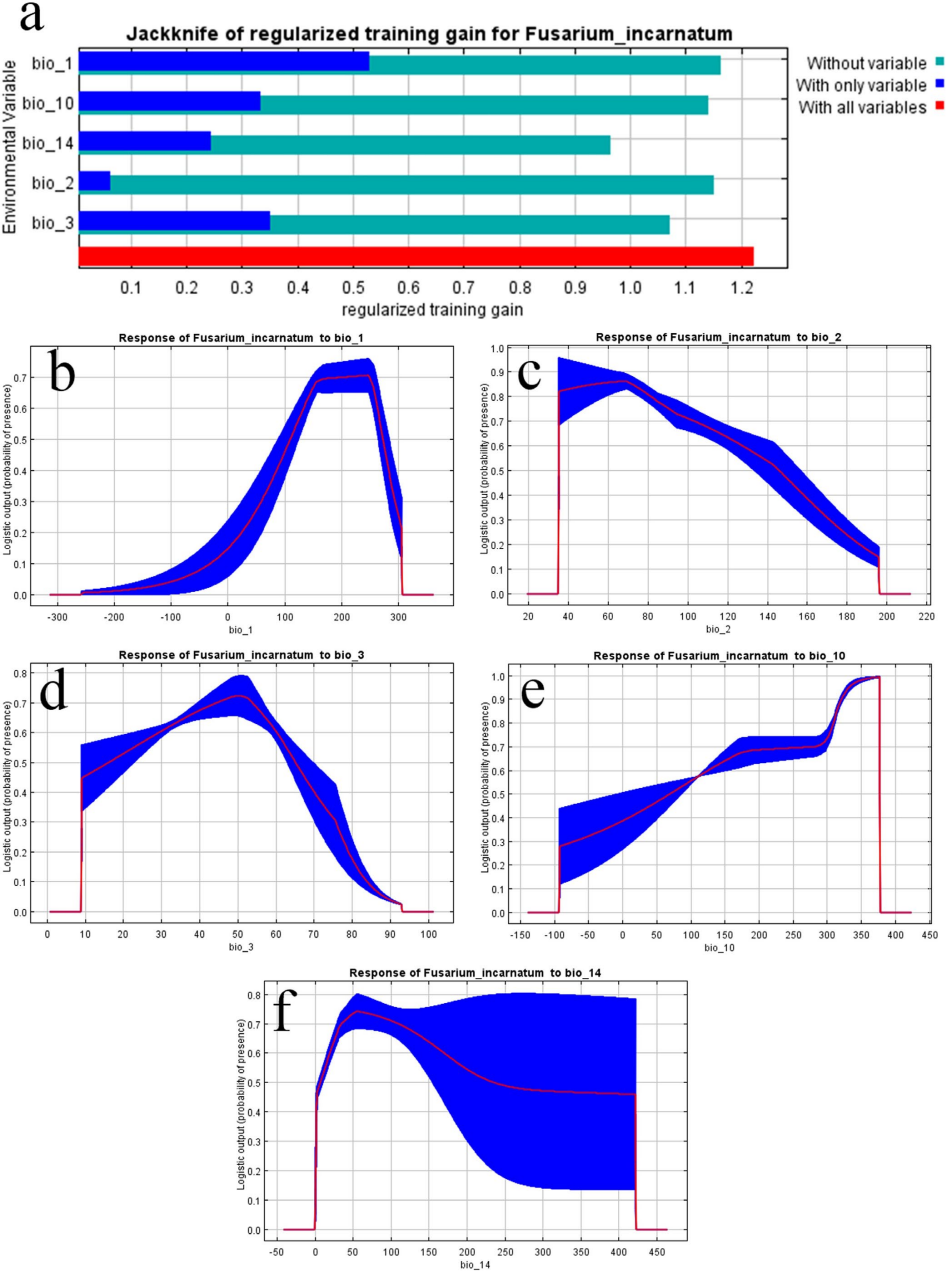
Response curves and jackknife test for bioclimatic variables. (a) Jackknife test showing regularized training gain when variables are used in isolation (dark blue bars) and when each variable is omitted (light blue bars), with the red line indicating total gain with all variables; (b–f) Response curves showing predicted probability of suitability (y‐axis) across the range of each bioclimatic variable (x‐axis): (b) Bio_1 (Annual Mean Temperature, °C); (c) Bio_2 (Mean Diurnal Range, °C); (d) Bio_3 (Isothermality, dimensionless index); (e) Bio_10 (Mean Temperature of Warmest Quarter, °C); (f) Bio_14 (Precipitation of Driest Month, mm). Error bars in panel (a) represent standard deviation across 10‐fold cross‐validation replicates.

Jackknife analysis (Figure 2a) revealed the relative importance of different bioclimatic variables in determining the distribution of *F. incarnatum*, with bio_1 (Annual Mean Temperature) identified as the dominant factor, followed by bio_2 (Mean Diurnal Range), bio_3 (Isothermality), bio_10 (Mean Temperature of Warmest Quarter), and bio_14 (Precipitation of Driest Month). The response curves (Figure 2b–f) showed specific ecological preferences for each variable: Bio_1 exhibited a bell‐shaped response with optimal suitability between 10°C and 20°C; bio_2 showed a declining response, indicating preference for lower diurnal temperature ranges; bio_3 displayed moderate tolerance for isothermality, with peak suitability around 40–50; bio_10 revealed a sharp increase in suitability above 30°C; and bio_14 presented a complex response with an initial increase followed by gradual decline, suggesting optimal precipitation requirements during dry periods. These response patterns collectively define the fundamental niche of *F. incarnatum* and explain its current global distribution patterns.

**FIGURE 3:**
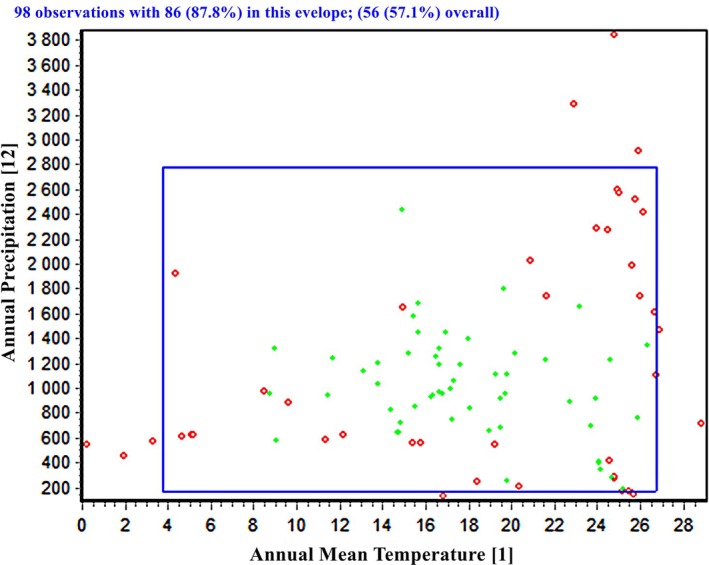
The envelope test for Bio_1 (Annual Mean Temperature, °C) and Bio_12 (Annual Precipitation, mm). Red points = occurrences outside bioclimatic envelope; green points = occurrences inside envelope. Box boundaries represent the 5th and 95th percentiles of occurrence data for Bio_1 and Bio_12; whiskers extend to 1.5× interquartile range.

The bioclimatic envelope analysis (Figure 3) of Annual Mean Temperature (bio_1) and Annual Precipitation (bio_12) provided crucial insights into the realized niche of *F. incarnatum*, with 58 observations, of which 55 (87.8%) fell within the defined envelope, representing 57.1% of the total dataset. The envelope plot demonstrated that the pathogen occupies a distinct climatic niche characterized by annual mean temperatures ranging approximately from 5°C to 25°C and annual precipitation levels between 200 and 2800 mm, with most occurrences concentrated in areas of moderate temperature and precipitation. This bioclimatic envelope defines the fundamental climatic requirements of *F. incarnatum* and serves as a crucial tool for predicting potential distribution boundaries, identifying climatically suitable areas for pathogen establishment, and understanding the environmental limitations that govern its ecological expansion under changing climatic conditions.

### 3.2 Current Distribution Modeling

#### 3.2.1 MaxEnt Model Results

**FIGURE 4:**
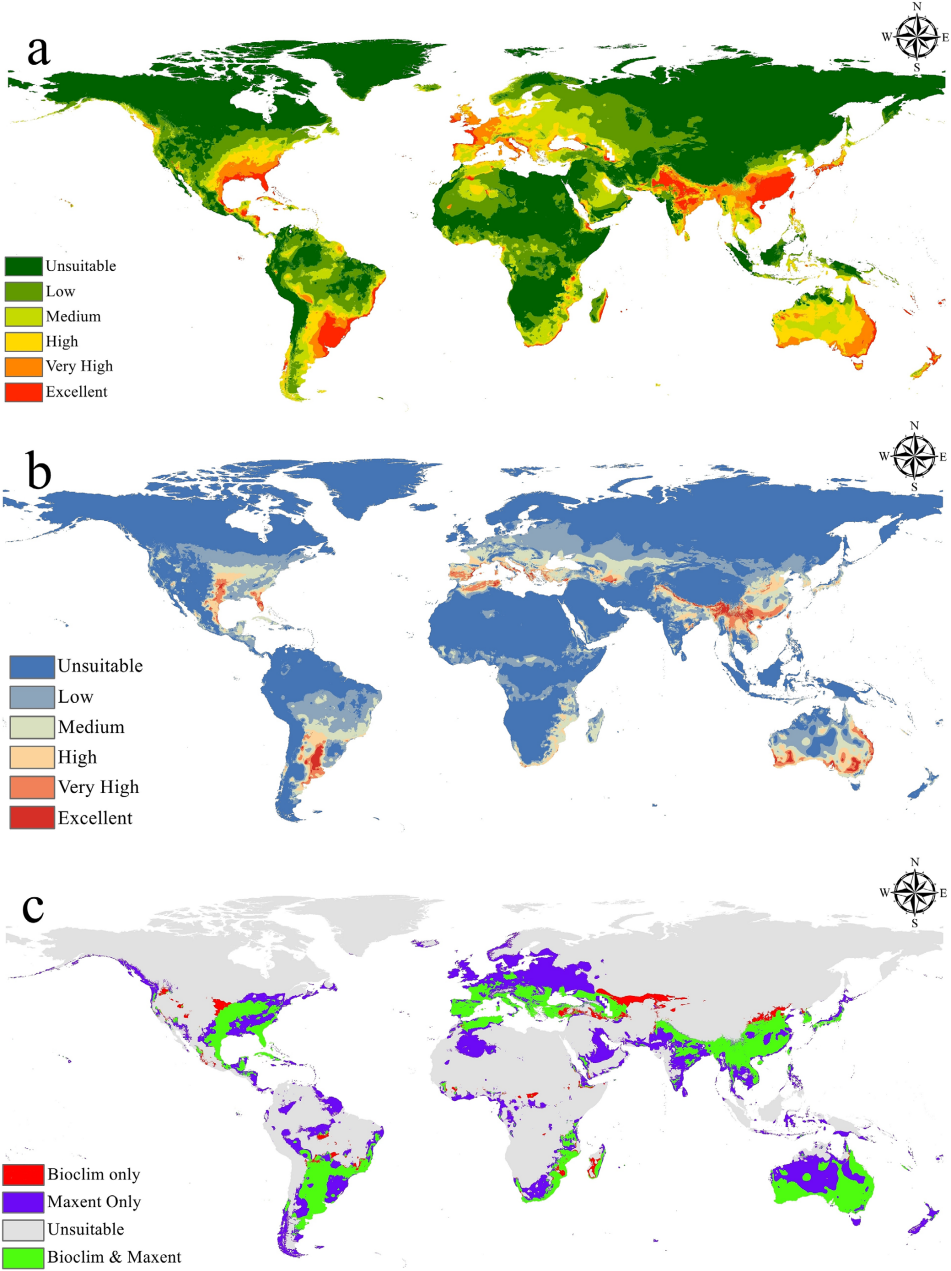
Current habitat suitability for *Fusarium incarnatum*. (a) MaxEnt model output showing relative probability of occurrence (0–1) classified into five suitability categories; (b) Bioclim model output showing areas meeting the species' climatic envelope criteria; (c) Consensus comparison map showing areas of agreement (green = both models predict suitable habitat; gray = both predict unsuitable; red = suitable only in Bioclim; purple = suitable only in MaxEnt).

The MaxEnt model predicted extensive areas of suitable habitat for *F. incarnatum* across multiple continents (Figure 4a), with habitat suitability classifications ranging from unsuitable to excellent. The model identified several key regions with high to excellent suitability, particularly within temperate and subtropical zones. In North America, the central United States, portions of Mexico, and southeastern regions showed high to very high suitability, with some areas achieving excellent suitability ratings. The model predicted moderate to high suitability across much of Europe, particularly in Mediterranean regions and parts of Eastern Europe.

The MaxEnt model revealed extensive suitable habitat throughout China, India, and Southeast Asia, predicting very high to excellent suitability for large portions of these regions. The model also identified suitable habitat in parts of the Middle East and Central Asia. For Africa, the model predicted variable suitability, showing moderate to high suitability in North Africa and parts of East Africa, while central and southern regions showed primarily low to moderate suitability. The model demonstrated considerable suitability along the western coast of South America and in parts of Brazil and Argentina. Australia showed moderate to high suitability in coastal regions, particularly in the eastern and southern portions of the continent.

#### 3.2.2 Bioclim Model Results

The Bioclim model produced a more conservative prediction of suitable habitat compared to the MaxEnt model (Figure 4b), classifying most of the global land area as unsuitable for *F. incarnatum*. The Bioclim approach identified substantially smaller areas of suitable habitat, with most suitable regions confined within specific climatic zones. Major areas of high to excellent suitability were predicted in parts of Europe, particularly within Mediterranean and temperate zones, along with specific locations in Asia, including portions of China and India.

The Bioclim model predicted limited suitable habitat in North America, primarily in the central United States and parts of Mexico, with considerably fewer areas of high suitability compared to the MaxEnt model. In South America, the model similarly indicated suitable habitats in restricted areas, particularly within the temperate regions of Argentina and southern Brazil. The Bioclim model showed minimal suitable habitat in Africa, with isolated pockets of suitability in North Africa and certain parts of East Africa. Australia showed moderate suitability in limited coastal areas, predominantly in the southern and southwestern regions.

#### 3.2.3 Comparative Analysis and Consensus Assessment

Comparative analysis of the MaxEnt and Bioclim models revealed substantial differences in their predictions (Figure 4c), highlighting the importance of using multiple modeling approaches for species distribution assessment. The comparison map delineated four distinct categories: areas deemed suitable only by Bioclim (red), areas considered suitable only by MaxEnt (purple), areas classified as unsuitable by both models (gray), and consensus areas where both models agreed on suitable habitat (green).

The consensus areas (green regions) represent the most reliable predictions for *F. incarnatum* distribution, as they are supported by both modeling approaches. These consensus areas were primarily located in temperate parts of Europe, certain portions of Asia including China and India, limited areas in North America, and specific locations in South America and Australia. The consensus suitable habitat comprised approximately 15%–20% of the areas identified as suitable by either model independently, indicating moderate agreement between the two approaches.

Areas identified as suitable exclusively by MaxEnt (purple areas) were extensive and included large portions of North America, Asia, Africa, and Australia. This pattern suggests that the MaxEnt model may be more liberal in its predictions, potentially identifying areas with marginally suitable conditions that the more conservative Bioclim model deemed unsuitable. Conversely, areas identified as suitable only by Bioclim (red areas) were relatively limited and scattered, predominantly occurring in specific climatic zones across Europe, Asia, and small portions of other continents.

The substantial differences between the two models underscore the importance of considering multiple modeling approaches when analyzing species distribution patterns. MaxEnt provides a comprehensive view of potential habitat suitability by accounting for complex interactions among environmental variables, while Bioclim offers a more conservative assessment based on the species' direct climatic envelope. The consensus areas identified by both models likely represent core suitable habitat for *F. incarnatum*, while areas of disagreement suggest locations where the species' establishment potential may be more uncertain and dependent on specific environmental factors or dispersal limitations.

These results establish a comprehensive baseline for understanding the current global distribution potential of *F. incarnatum* and provide a foundation for evaluating future distribution changes under climate change scenarios. Identification of consensus regions is particularly important for prioritizing monitoring efforts and developing targeted management strategies for this economically important fungal pathogen.

### 3.3 Distribution of *Fusarium incarnatum* Under Climate Change: Projections for 2050 and 2070

Climate change projections indicate substantial alterations in the global range and ecological suitability for *Fusarium incarnatum*, an economically important plant pathogen (Table 1). This analysis examines four climate scenarios: moderate mitigation (RCP 2.6) and high emission (RCP 8.5) pathways for 2050 and 2070, providing critical insights for plant disease management and agricultural planning strategies.

**TABLE 1:** Projected changes in global habitat suitability of *Fusarium incarnatum* under four climate change scenarios.

Habitat category	Current (baseline)	2050 RCP 2.6	2050 RCP 8.5	2070 RCP 2.6	2070 RCP 8.5
Stable suitable (%)	—	11.2	10.8	10.5	9.6
Habitat gain (%)	—	3.4	4.9	4.1	7.3
Habitat loss (%)	—	−1.8	−2.1	−1.5	−2.7
Stable unsuitable (%)	—	85.2	84.2	84.9	80.8
Net change (%)	—	+1.6	+2.8	+2.6	+4.6
Total suitable area (%)	~12.6	14.6	15.7	14.6	16.9

*Note:* Values are visual estimates derived from proportional analysis of (Figure 5) color coverage across the four scenario maps. Percentages refer to global land surface area.

**FIGURE 5:**
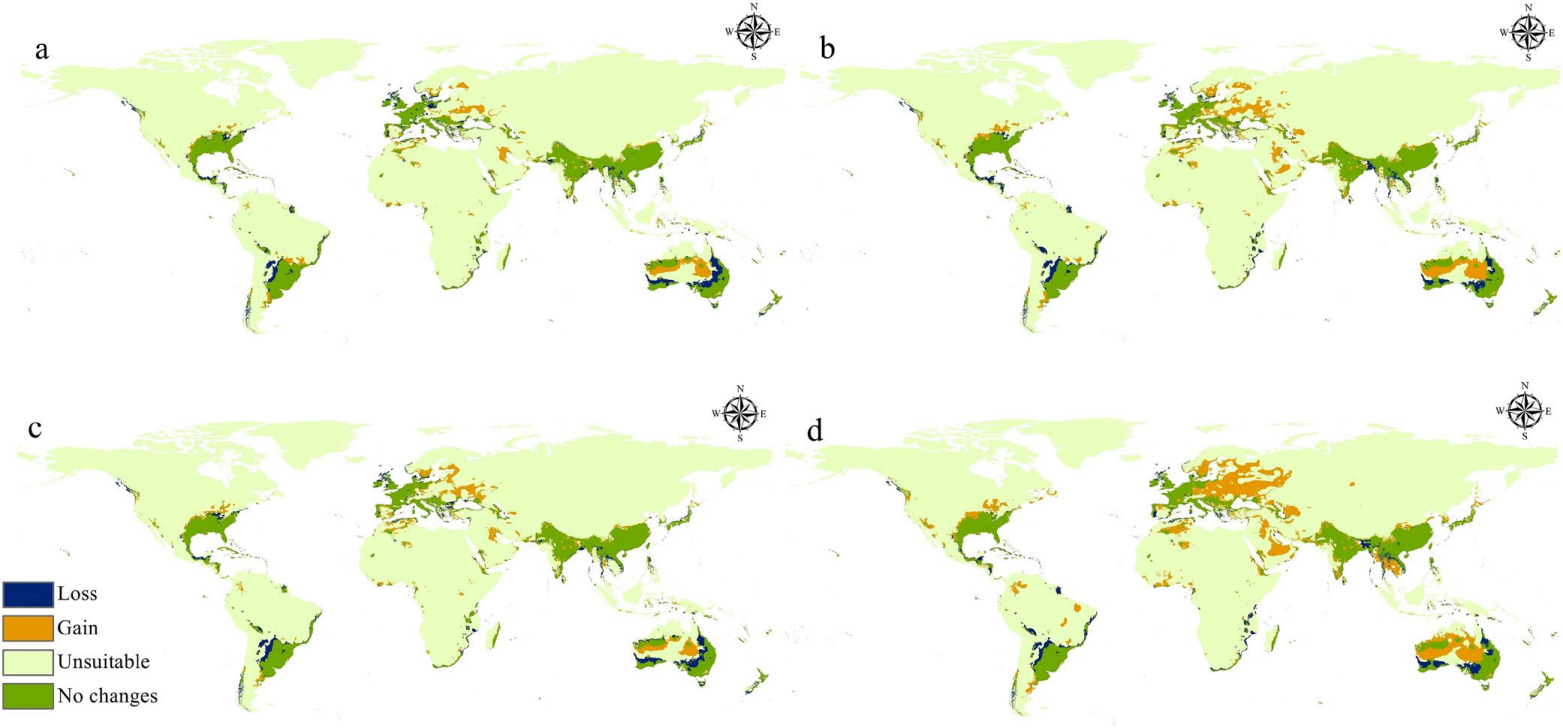
Calibration maps showing projected changes in range of *Fusarium incarnatum*. (a) RCP 2.6, 2050; (b) RCP 8.5, 2050; (c) RCP 2.6, 2070; (d) RCP 8.5, 2070. Colors indicate: stable suitable habitat (dark green = no change in suitable status), habitat gain (orange = transition from unsuitable to suitable), habitat loss (dark blue = transition from suitable to unsuitable), and stable unsuitable habitat (light green = no change in unsuitable status).

#### 3.3.1 Projected Changes in Pathogen Distribution

##### 3.3.1.1 2050 Climate Scenarios

###### 3.3.1.1.1 RCP 2.6 Scenario (Moderate Mitigation) (Figure 6a)

**FIGURE 6:**
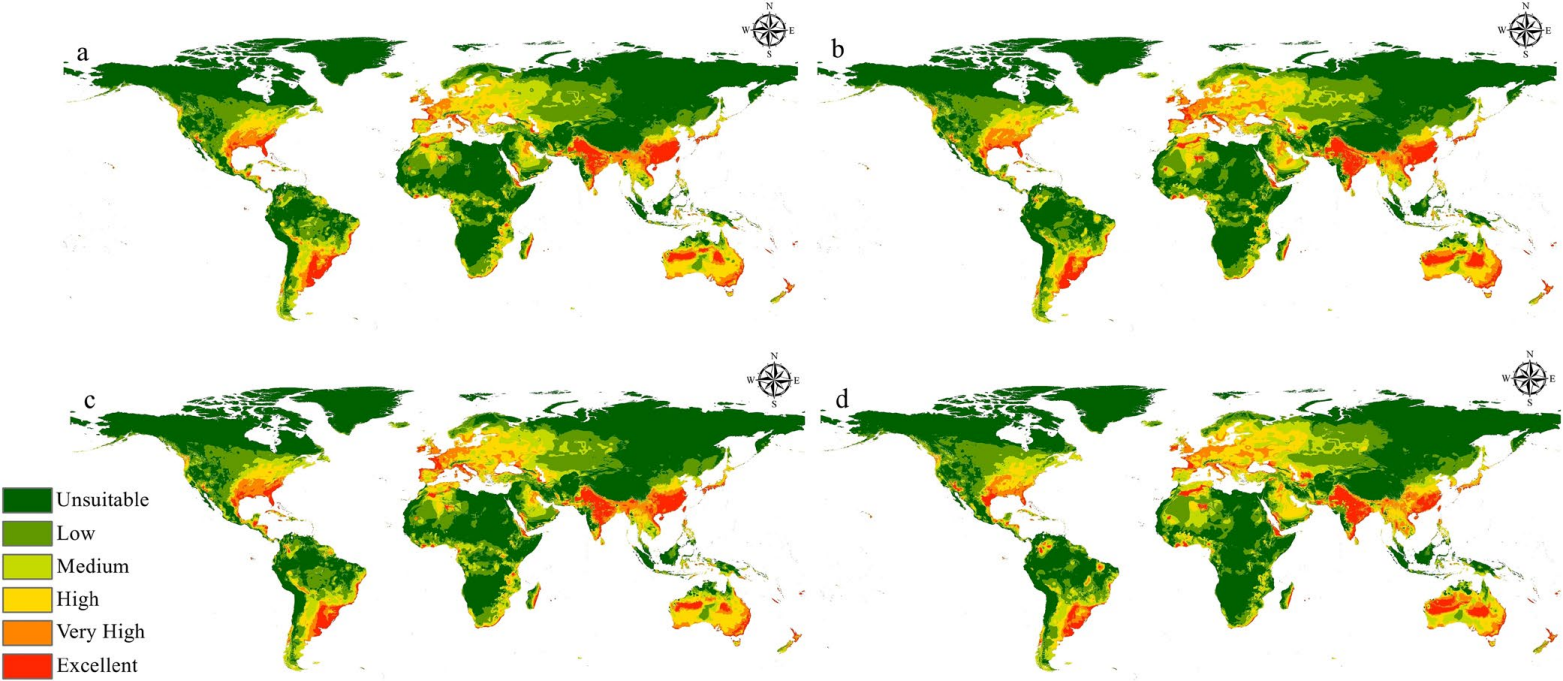
Predicted future distribution of habitat suitability of *Fusarium incarnatum* under climate change scenarios. (a) RCP 2.6 emission scenario for 2050 (2041–2060); (b) RCP 8.5 emission scenario for 2050 (2041–2060); (c) RCP 2.6 emission scenario for 2070 (2061–2080); (d) RCP 8.5 emission scenario for 2070 (2061–2080). Projections based on ensemble means of three GCMs (BCC‐CSM1.1, CCSM4, MRI‐CGCM3). Color scale: unsuitable (0–0.2), low (0.2–0.4), medium (0.4–0.6), high (0.6–0.8), very high (0.8–1.0).

Under the moderate mitigation scenario, *F. incarnatum* exhibits concerning poleward expansion by 2050. Northern regions, including Canada, northern Europe, and Russia, transition from unsuitable to medium and high suitability zones, suggesting probable establishment of the pathogen in previously unaffected areas. Current hotspots in sub‐Saharan Africa, portions of South Asia, and the Middle East show high to excellent suitability conditions, indicating sustained disease pressure in these regions. The pathogen's suitability remains relatively stable across tropical regions of South America and Southeast Asia.

###### 3.3.1.1.2 RCP 8.5 Scenario (High Emissions) (Figure 6b)

The high emissions trajectory accelerates the poleward expansion of suitable habitat for *F. incarnatum*. By 2050, extensive areas of Canada, Scandinavia, and Siberia exhibit high to excellent suitability, indicating substantial range expansion. This expansion is accompanied by increased suitability in already affected regions, particularly in Africa and parts of Asia, where optimal conditions become increasingly prevalent. Australia shows variable patterns, with enhanced suitability in northern regions, though some southern areas experience reduced risk levels.

##### 3.3.1.2 2070 Climate Projections

###### 3.3.1.2.1 RCP 2.6 Scenario (Sustained Moderate Mitigation) (Figure 6c)

By 2070, even under moderate mitigation efforts, the pathogen's suitable habitat continues expanding poleward. Boreal regions of Canada and Russia show increased suitability, while traditional high‐risk areas in Africa and Asia continue experiencing substantial disease pressure. The Mediterranean region shows elevated risk, suggesting potential impacts on European agriculture. This scenario maintains some degree of stability in tropical regions, though with gradual increases in suitability.

###### 3.3.1.2.2 RCP 8.5 Scenario (Sustained High Emissions) (Figure 6d)

The 2070 high emissions scenario demonstrates the most dramatic transformation in *F. incarnatum* distribution. Vast areas of the northern hemisphere exhibit excellent suitability, encompassing much of Canada, the northern United States, and northern Eurasia. This represents a substantial increase in potential disease risk for agricultural regions that have historically been protected by climatic barriers. Simultaneously, many currently affected regions maintain or intensify their disease burden, resulting in global expansion rather than mere redistribution.

### 3.4 Limiting Factor Map

**FIGURE 7:**
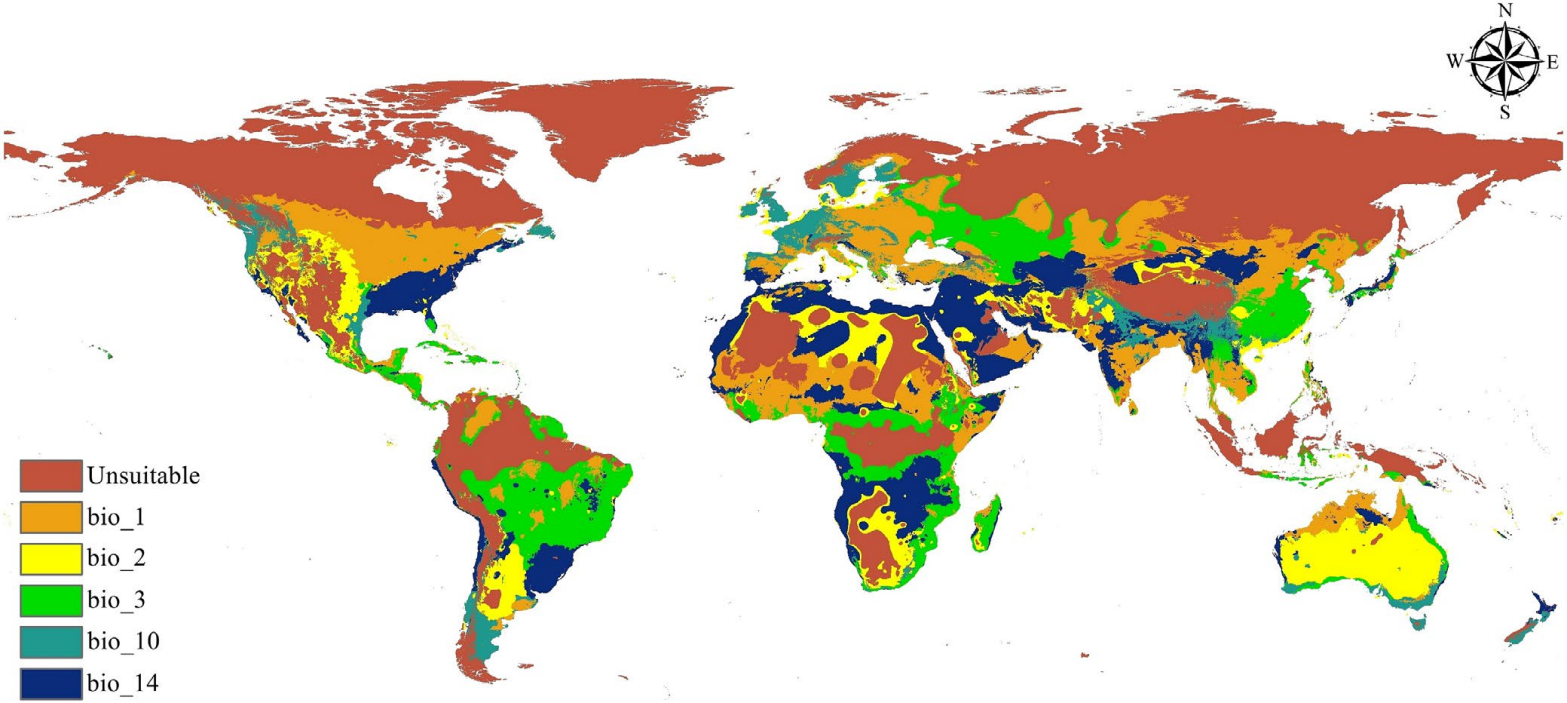
Limitation factor map identifying the primary bioclimatic constraints on *Fusarium incarnatum* distribution across different geographic regions. Colors indicate which variable most strongly limits suitability: Bio_1 (Annual Mean Temperature), Bio_2 (Mean Diurnal Range), Bio_3 (Isothermality), Bio_10 (Mean Temperature of the Warmest Quarter), or Bio_14 (Precipitation of the Driest Month).

The bioclimatic limiting factor analysis (Figure 7) reveals distinct regional patterns of environmental constraints governing the global distribution of *Fusarium incarnatum*. Temperature‐related variables (bio_1—Annual Mean Temperature and bio_2—Mean Diurnal Range) are identified as primary limiting factors across extensive northern regions, including Canada, northern Russia, and Arctic areas, indicating that insufficient thermal conditions fundamentally prevent pathogen establishment in these localities. The pathogen's inability to colonize these areas stems from inadequate annual heat accumulation for fungal development, excessive diurnal temperature fluctuations exceeding physiological tolerances, and abbreviated growing seasons that prevent completion of infection cycles. Central Asian regions and portions of Eastern Europe are predominantly limited by bio_3 (Isothermality), suggesting that *F. incarnatum* is particularly sensitive to extreme seasonal temperature variations and erratic thermal regimes that disrupt fungal life cycles and prevent stable pathogen establishment.

Moisture‐related limitations dominate in tropical and subtropical regions, where bio_10 (Mean Temperature of the Warmest Quarter) and bio_14 (Precipitation of the Driest Month) serve as principal constraining factors across various parts of Africa, South America, and Asia. The bio_10 constraints demonstrate that elevated temperatures during the warmest quarter exceed the pathogen's thermal tolerance, causing thermal stress that reduces spore viability and disrupts infection processes. Conversely, bio_14 limitations highlight the pathogen's dependence on continuous moisture, as insufficient precipitation during critical dry periods inhibits spore germination, infection cycles, and pathogen survival during extended dry seasons. Moisture limitations are most pronounced in semi‐arid regions, where the lack of sustained humidity necessary for successful infection creates natural barriers to pathogen establishment and spread.

The complex mosaic of limiting factors across different geographic regions provides critical insights into *F. incarnatum*'s ecological requirements and potential dispersal boundaries under changing climatic conditions. Transition zones between different bioclimatic constraints, particularly in Mediterranean regions, mountainous areas, and coastal zones, illustrate the intricate interplay of multiple environmental factors and pathogen distribution. Understanding these limitation patterns is crucial for predicting how climate change might alter the geographic distribution of the pathogen, as shifting temperature and precipitation patterns could potentially overcome existing bioclimatic barriers, allowing *F. incarnatum* to expand into previously unsuitable areas. This analysis emphasizes the importance of monitoring bioclimatic variables as early warning indicators for future disease range expansions and the need for adaptive management strategies in regions where existing constraints may be weakening due to climate change.

## 4 Discussion

The present study forms the first work that discussed how the global warming will change the distribution pattern of *Fusarium incarnatum especially on the global scale*. A critical caveat that must be acknowledged upfront is that the GBIF occurrence data used in this study are primarily based on ITS rDNA identification, which cannot reliably distinguish among the more than 30 cryptic phylogenetic species (phylo‐species) within the *Fusarium incarnatum‐equiseti* species complex (FIESC; O'Donnell et al. 2013, 2020). Consequently, the modeled ecological niche represents an aggregate distribution for the FIESC rather than a single taxonomic entity, and the bioclimatic envelope characterized here likely encompasses the combined climatic tolerances of multiple cryptic lineages. This taxonomic aggregation means that the predicted suitable habitat may be broader than that of any single FIESC member. Future modeling efforts incorporating phylogenetically verified, multi‐locus occurrence data would substantially refine these predictions. Our findings provide compelling evidence of climate change's profound influence on the global distribution of *Fusarium incarnatum*, revealing substantial range expansions and shifts in habitat suitability patterns under projected warming scenarios. The MaxEnt modeling approach employed here achieved excellent predictive performance (AUC = 0.88, TSS = 0.80), successfully identifying key bioclimatic variables driving the pathogen's distribution and projecting alarming poleward expansions under both moderate and high emission scenarios. These results align with recent research on related *Fusarium* species, particularly *F. oxysporum*, which has demonstrated cosmopolitan distribution and enhanced virulence under changing climatic conditions. The robust performance metrics obtained in our study are consistent with previous applications of MaxEnt modeling for plant pathogen distribution, where the software has proven effective for forest pathogens that have achieved similarly high AUC values (Tang et al. 2021).

The identification of Annual Mean Temperature (bio_1) as the primary limiting factor for *F. incarnatum* distribution provides crucial insights into the mechanistic basis of climate‐driven range shifts. The bell‐shaped response curve indicating optimal suitability between 10°C and 20°C suggests that this fungus occupies a specific thermal niche that could expand considerably under global warming conditions. This temperature dependence is consistent with broader patterns observed in *Fusarium* species, where temperature fluctuations associated with climate change have been linked to increased disease severity and altered host‐pathogen interactions (Chakraborty et al. 2000; Garrett et al. 2006). The secondary importance of Mean Diurnal Range (bio_2) and precipitation variables (bio_14) indicates that *F. incarnatum* requires stable temperature conditions and adequate moisture for optimal establishment, similar to observations reported for other fungal pathogens, where thermal stability and moisture availability are critical determinants of infection success (Chakraborty et al. 2011; Elad and Pertot 2014).

The substantial differences between MaxEnt and Bioclim model predictions observed in our study reflect well‐documented methodological differences in niche approximation, not model inadequacy. MaxEnt employs a machine‐learning algorithm that captures complex, non‐linear interactions among environmental variables, while Bioclim applies a simple climate envelope approach based on extreme values of occurrence localities. Divergence between these approaches is expected and informative: consensus areas represent the most robust predictions, while areas of disagreement highlight uncertainty that warrants further investigation (Araújo and New 2007; Thuiller et al. 2009). This dual‐model approach is standard practice in species distribution modeling and enhances the reliability of predictions by identifying areas where different modeling philosophies converge.

The projected poleward expansion of *F. incarnatum* under both RCP 2.6 and RCP 8.5 scenarios poses substantial threats to agricultural systems in previously unaffected regions. Our models predict that extensive areas of Canada, northern Europe, and Siberia will transition from unsuitable to medium‐high suitability zones by 2050–2070, potentially exposing novel agricultural production areas to this economically significant pathogen. These range shift projections are consistent with broader trends in plant pathogen responses to climate change, where pathogen distributions are shifting to enable disease establishment in novel regions (Bebber et al. 2013; Chaloner et al. 2021). The pronounced expansion observed under the RCP 8.5 scenario emphasizes the critical need for climate mitigation policies to limit pathogen spread. Similar poleward expansions have been documented for various plant diseases, including pine wilt disease in China, where MaxEnt modeling revealed substantial habitat increases in northern regions under climate change projections (Tang et al. 2021; Ikegami and Jenkins 2018).

The comparative analysis of MaxEnt and Bioclim models yielded valuable insights into methodological considerations for disease distribution modeling. MaxEnt predicted more extensive suitable habitats, demonstrating its ability to capture complex environmental interactions, while the Bioclim model produced more conservative projections based on direct climate envelopes. The consensus regions identified by both models, with approximately 15%–20% overlap, represent the most reliable predictions for current *F. incarnatum* distribution and should be prioritized for monitoring and management efforts. This methodological comparison underscores the value of ensemble modeling approaches in species distribution research, particularly for pathogens where accurate predictions are essential for disease management decisions (Araújo and New 2007; Thuiller et al. 2009). The differences between models highlight inherent uncertainties in distribution modeling, suggesting that management decisions should incorporate multiple modeling approaches and focus on consensus areas for the most reliable predictions.

Our projections of climate‐mediated range expansion for *F. incarnatum* align with emerging evidence from other fungal pathogen systems. Recent studies on wheat fungal diseases have documented increasing *Fusarium* incidence correlating with warming trends, particularly in regions experiencing altered precipitation patterns (Vaughan et al. 2016; West et al. 2012). The wheat pathosystem, in particular, has shown vulnerability to *Fusarium* head blight expansion into higher latitudes, with significant economic implications for northern grain‐producing regions (McMullen et al. 2012; Miedaner and Juroszek 2021). These observations suggest that our predictive models may already be manifesting in real‐world agricultural systems, lending credence to our projections of continued expansion under future climate scenarios.

The bioclimatic envelope and limiting factor analyses provide essential frameworks for establishing early warning systems and targeted surveillance programs for *F. incarnatum*. The identification of temperature‐related limitations in northern latitudes and moisture constraints in semi‐arid regions offers specific targets for monitoring climate threshold changes that may trigger pathogen establishment. The projected transition zones, particularly in Mediterranean regions and coastal areas, represent critical locations where changing climatic conditions may overcome existing bioclimatic barriers, facilitating rapid pathogen expansion. These findings have immediate practical applications for plant quarantine services, agricultural extension programs, and crop protection strategies, enabling proactive rather than reactive management approaches. The habitat suitability maps generated here can inform risk assessment frameworks, guide resource allocation for disease surveillance, and support the development of climate‐resilient crop varieties in regions projected to experience increased pathogen pressure (Bebber 2015; Caubel et al. 2015).

The implications of our findings extend beyond *F. incarnatum* to address broader concerns about climate change impacts on plant pathogen communities and global food security. The projected range expansions align with documented increases in *Fusarium* occurrences on wheat kernels attributed to climate change, suggesting our predictions may already be evident in agricultural systems (Dahl and Wilson 2018; Moretti et al. 2019). The identification of emerging risk zones in major food‐producing regions, including the northern Great Plains of North America and the grain belts of Russia and Eastern Europe, underscores the urgent need for adaptive management strategies and climate‐resilient agricultural practices.

The mycotoxin production potential of *F. incarnatum* adds another dimension to the public health implications of its projected range expansion. Members of the FIESC complex are known producers of various mycotoxins, including moniliformin, fusaric acid, and beauvericin, which pose significant health risks to humans and animals through contaminated grain products (Moretti et al. 2019; Busman et al. 2018). As the pathogen expands into novel agricultural regions, previously unexposed crop production systems may lack the infrastructure, expertise, and regulatory frameworks necessary to manage mycotoxin contamination effectively (van der Fels‐Klerx et al. 2012; Streit et al. 2013). This concern is particularly acute for northern regions where regulatory systems and quality control measures for mycotoxins in grain products may be less developed due to historically low disease pressure from *Fusarium* species.

The host range versatility of *F. incarnatum* further complicates its management under changing climatic conditions. This pathogen has been documented infecting numerous economically important crops, including cereals, legumes, vegetables, and ornamental plants, demonstrating remarkable adaptability across diverse agricultural systems (Summerell and Leslie 2011; Leslie and Summerell 2006). Recent investigations have revealed its pathogenicity on emerging crops and in novel host‐pathogen combinations, suggesting that climate‐driven range expansion may facilitate interactions with previously unexposed host species (O'Donnell et al. 2020).

Despite the robust methodology employed in this study, several limitations warrant consideration. The reliance on presence‐only data from GBIF may introduce sampling bias toward well‐surveyed regions, potentially underestimating the pathogen's occurrence in less‐studied areas. The spatial rarefaction to 112 occurrence points, while necessary for reducing spatial autocorrelation, may have eliminated ecologically relevant occurrences and reduced the model's ability to capture fine‐scale distribution patterns. Furthermore, our models assume unlimited dispersal capacity and do not account for host plant distributions, agricultural practices, or biotic interactions that could influence pathogen establishment in climatically suitable regions. The use of only five bioclimatic variables, while addressing multicollinearity, may have excluded other ecologically relevant factors such as soil properties, ultraviolet radiation exposure, or seasonal climatic extremes that could affect pathogen survival and dispersal.

A key limitation of the present study is that climatic suitability alone does not equate to agricultural risk. The establishment and impact of *F. incarnatum* on crops depends not only on favorable climate but also on the availability of susceptible host species and the structure of agricultural systems in newly suitable regions. Future studies should overlay our habitat suitability maps with high‐resolution crop distribution datasets—such as SPAM (Spatial Production Allocation Model) and EarthStat—to generate crop‐specific risk assessments for major cereal, legume, and vegetable production systems. Such integration would enable targeted management recommendations for regions projected to experience increased pathogen pressure and would substantially improve the applied value of climate‐based distribution models for fungal plant pathogens.

From an applied agricultural perspective, this taxonomic aggregation remains ecologically meaningful because: (1) multiple FIESC phylo‐species share similar pathogenic potentials and host ranges; (2) current diagnostic capabilities in most agricultural settings still rely on morphological or ITS‐based identification; and (3) management strategies for FIESC members are broadly similar. Nevertheless, future modeling efforts incorporating phylogenetically verified occurrence data would refine these predictions.

The high variability in bio_14 (Precipitation of Driest Month) contribution across cross‐validation folds reflects the heterogeneous moisture regimes across *F. incarnatum*'s global distribution. While this variable shows higher uncertainty than temperature variables, its ecological relevance to fungal pathogen survival during dry periods justifies retention. Future models incorporating soil moisture or relative humidity data may reduce this uncertainty.

The temporal dynamics of pathogen spread represent another important consideration not fully captured by our static modeling approach. Disease establishment in newly suitable areas depends not only on climatic suitability but also on propagule pressure, dispersal mechanisms, and the availability of susceptible hosts (Lockwood et al. 2005; Simberloff 2009). *Fusarium* species exhibit multiple dispersal pathways, including wind‐borne ascospores, water splash dispersal of macroconidia, movement on infected plant material and seeds, and anthropogenic transport through agricultural trade (Burgess and Liddell 1983; Goswami and Kistler 2004). The relative importance of these dispersal mechanisms may vary across different geographic regions and agricultural systems, influencing the rate at which newly suitable habitats are colonized. Integration of dispersal models with habitat suitability predictions would provide more realistic projections of disease spread dynamics.

Future research should integrate host plant distribution data and agricultural land use patterns to enable more realistic assessment of actual disease risk beyond simple climatic suitability. High‐resolution crop distribution datasets (e.g., SPAM, EarthStat) could be overlaid with our climatic suitability maps to generate crop‐specific risk assessments for major food production systems. This would enable targeted management recommendations for regions projected to experience increased pathogen pressure.

The interaction between climate change and agricultural intensification presents additional complexities for predicting future disease distributions. Agricultural expansion into marginal lands, changes in cropping patterns, and adoption of new cultivars in response to changing climatic conditions may alter host availability and susceptibility in ways that either facilitate or constrain pathogen establishment (Gregory et al. 2009; Juroszek and von Tiedemann 2013). Monoculture systems and reduced crop diversity, increasingly common in intensive agricultural landscapes, may provide more favorable conditions for *Fusarium* diseases by reducing ecological complexity and eliminating natural biological controls (Tilman et al. 2002; Bever et al. 2015). Conversely, implementation of integrated pest management strategies, crop rotation, and resistant varieties could mitigate disease impacts even in climatically suitable regions.

The potential for evolutionary adaptation in *F. incarnatum* populations in response to changing climatic conditions represents an additional source of uncertainty in long‐term projections. Fungal pathogens exhibit considerable phenotypic plasticity and rapid evolutionary potential, with documented cases of adaptation to novel environmental conditions, host species, and management practices occurring over relatively short timescales (McDonald and Stukenbrock 2016; Stukenbrock and McDonald 2008). Experimental evolution studies on *Fusarium* species have demonstrated their capacity for rapid thermal adaptation and increased virulence under selection pressure (Schoustra et al. 2007; Seidl and Thomma 2014). Climate change may therefore not only alter the geographic distribution of existing pathogen populations but also drive evolutionary changes that enhance their fitness and pathogenic capacity in novel environments.

Our findings underscore the critical importance of integrating climate change considerations into plant biosecurity and disease management frameworks. Traditional approaches to plant disease management have typically focused on reactive measures implemented after disease establishment, but the projected rapid expansion of *F. incarnatum* into novel regions necessitates more proactive strategies (Bebber et al. 2013; Brasier 2008). These should include enhanced surveillance in predicted risk zones, development of early warning systems based on climatic triggers, establishment of quarantine measures to prevent anthropogenic dispersal, and investment in breeding programs for resistant varieties adapted to changing disease pressures. International cooperation and data sharing will be essential for effective management of this cosmopolitan pathogen as it continues to expand its range under climate change.

The economic implications of expanded *F. incarnatum* distribution are substantial. *Fusarium* diseases collectively cause billions of dollars in annual crop losses globally, with costs accruing from reduced yields, quality downgrades due to mycotoxin contamination, increased fungicide applications, and trade restrictions on contaminated products (Xu et al. 2008; Wegulo et al. 2015). The expansion of this pathogen into currently low‐risk regions could impose significant new economic burdens on agricultural systems that lack the infrastructure and expertise to manage *Fusarium* diseases effectively. Economic impact assessments should be integrated with bioclimatic modeling to inform policy decisions regarding climate change mitigation, agricultural adaptation strategies, and allocation of research resources for disease management.

Future research directions should focus on several key areas to enhance our understanding and predictive capacity for *F. incarnatum* distribution under climate change. First, incorporation of host plant distribution data and agricultural land use patterns would enable more realistic assessment of actual disease risk beyond simple climatic suitability. Future studies should integrate host crop distribution data (e.g., from SPAM or EarthStat databases) with our climatic suitability maps to generate crop‐specific risk assessments. This would enable targeted management recommendations for major food production systems in newly suitable regions. Second, experimental studies examining the physiological responses of *F. incarnatum* to elevated temperatures, altered precipitation regimes, and extreme weather events would provide mechanistic understanding to complement correlative distribution models. Third, long‐term monitoring programs in predicted expansion zones would enable validation of model projections and provide early warning of actual range shifts. Fourth, investigation of interactions between *F. incarnatum* and other members of the soil microbiome under changing climatic conditions could reveal important biological constraints or facilitators of pathogen establishment. Finally, development of integrated models combining climate projections, pathogen distribution, host plant phenology, and agricultural management practices would provide more comprehensive tools for disease risk assessment and management planning.

The research presented here demonstrates the power of species distribution modeling combined with climate change projections for understanding potential shifts in plant pathogen distributions. Our findings for *F. incarnatum* likely reflect broader patterns affecting numerous fungal pathogens, suggesting that climate change will fundamentally reshape plant disease landscapes globally. The projected expansion of this economically important pathogen into northern latitudes, particularly major grain‐producing regions of North America and Eurasia, presents significant challenges for global food security in coming decades. Proactive management strategies informed by predictive modeling, enhanced surveillance systems, and international cooperation will be essential for mitigating the agricultural and economic impacts of climate‐driven pathogen range expansions. This study provides crucial baseline data and predictive frameworks to support evidence‐based decision‐making in plant health policy, biosecurity planning, and agricultural adaptation to climate change.

## 5 Conclusions

This study provides comprehensive evidence that climate change will significantly alter the global distribution of *Fusarium incarnatum*, with substantial implications for agricultural sustainability and food security. Our MaxEnt modeling approach successfully identified Annual Mean Temperature as the primary bioclimatic factor governing current distributions and predicted concerning poleward expansions under both moderate and high emission scenarios. The projected expansion of suitable habitat into northern regions of Canada, Europe, and Asia by 2050–2070 represents a significant emerging threat to agricultural systems in these areas, particularly under high emission trajectories. The identification of consensus suitable areas through comparative modeling provides reliable targets for prioritized monitoring and management efforts. The limiting factor analysis reveals distinct regional patterns of environmental constraints that will govern future range expansion dynamics. These findings emphasize the urgent need for proactive disease surveillance systems, climate‐resilient crop development, and international cooperation in plant biosecurity to address the challenges posed by climate‐driven pathogen range shifts. The methodological framework and predictive maps generated here provide essential tools for evidence‐based policy development and adaptive management strategies to protect global food security in a changing climate.

## Author Contributions

**Jawaher Ibrahim Alahadeb:** conceptualization (equal), data curation (equal), formal analysis (equal), funding acquisition (equal), investigation (equal), methodology (equal), software (equal), supervision (equal), validation (equal), visualization (equal), writing – original draft (equal), writing – review and editing (equal).

## Funding

This work was supported by the Deanship of Scientific Research, Majmaah University, Saudi Arabia (R‐2026‐291).

## Ethics Statement

This manuscript does not contain any studies with human participants or animals performed.

## Conflicts of Interest

The author declares no conflicts of interest.

## Data Availability

Species occurrence data were obtained from the Global Biodiversity Information Facility (GBIF) database (www.gbif.org; query: *Fusarium incarnatum*). Bioclimatic variables were obtained from the WorldClim database (www.worldclim.org; version 2.0, 2.5 arc‐minute resolution). Future climate projection data were obtained from WorldClim CMIP5 downscaled climate projections. All data sources are publicly available.
